# Editorial: From bench to clinic: novel vaccines and therapeutics – “success stories”

**DOI:** 10.3389/fcimb.2025.1701460

**Published:** 2025-11-06

**Authors:** Arundhathi Venkatasubramaniam, Nathan K. Archer

**Affiliations:** 1 Center for Vaccine Development, University of Maryland Baltimore, Baltimore, MD, United States; 2 Johns Hopkins Medicine, Johns Hopkins University, Baltimore, MD, United States

**Keywords:** vaccines, clinical trial, therapeutics, tuberculosis, COVID - 19, trypanosoma cruzi (T cruzi)

The commercialization of a vaccine requires several steps, from discovery and development to Phase I-III clinical trials (Guy and Barrere, 2011; Cunningham and Garçon, 2016; Singh and Mehta, 2016). We are pleased to present a Research Topic on Frontiers in Cellular and Infection Microbiology focusing on these aspects of vaccine development. This collection of manuscripts brings together a myriad of studies that look at vaccine engineering, dose effectiveness, the use of adjuvants, and the immune response elicited by vaccines. We extend our heartfelt gratitude to the authors, reviewers, and the dedicated editorial team who have contributed to the completion of this Research Topic.

The study “Engineering a dual vaccine against COVID-19 and tuberculosis” highlighted a novel vaccine against both diseases. This is especially significant considering that there is a lack of vaccines against tuberculosis other than BCG (Maani and Petersen, 2025). The article proposed the use of recombinant BCG strains as a platform for the development of safe and effective vaccines. Interesting future work could include booster vaccinations, testing the vaccine in mouse models (of both TB and COVID-19), evaluation of a freeze-dried formulation of the recombinant vaccine, and examining immune response in non-human primate models. 

The contribution “Vaccination with parasite-specific TcTASV proteins combined with recombinant baculovirus as a delivery platform protects against acute and chronic *Trypanosoma cruzi* infection” proved to be promising in delivering a vaccine candidate against *Trypanosoma cruzi*, the parasite that causes Chagas disease. The authors discussed a combination of parasite-specific proteins alongside a baculovirus platform used as a delivery, displaying one such protein on the capsid. This combination was successful, with vaccinated mice surviving a lethal *T. cruzi* challenge and presenting a reduced parasitic load in chronic infection. The success of the vaccine against other strains of *T. cruzi* and the durability of the immune response remain to be seen (Brenière and Waleckx, 2016).

Animal models are essential and functional during the discovery and development phases of vaccine creation, but eventually, all vaccines must be tested in humans. The articles “Effectiveness of two-dose vs. one-dose varicella vaccine in children in Shanghai, China: a prospective cohort study” and “TRAIL and IP-10 dynamics in pregnant women post COVID-19 vaccination: associations with neutralizing antibody potency” are both human studies, albeit different. The former is a clinical trial that compared the effectiveness of one vs two doses of the varicella vaccine in children in Shanghai, China, concluding that two doses are more effective than one. The latter studied the dynamics of biomarkers TRAIL and IP-10 for COVID-19 infection in pregnant women post vaccination, along with the inhibition of neutralizing antibodies against COVID-19 strains. These two studies are of special significance because they studied the course of vaccination in children and pregnant women, both vulnerable groups that merit additional analysis (Marshall and McMillan, 2016; Örtqvist, 2010).

Although the development of a successful vaccine represents a significant challenge, these articles all bring forward ways in which targets/biomarkers (Sohail Ahmed and Black, 2011) can be identified that enable scientists and clinicians the tools to develop vaccines and therapeutics. Finally, vaccine and therapeutic research is an exciting, rapidly evolving field that harnesses new technologies to help reduce the burden of several diseases or to eliminate them from our communities.
